# Mechanism of Cytosolic Phospholipase A_2_ Activation in Ghrelin Protection of Salivary Gland Acinar Cells against Ethanol Cytotoxicity

**DOI:** 10.1155/2010/269274

**Published:** 2010-06-22

**Authors:** Bronislaw L. Slomiany, Amalia Slomiany

**Affiliations:** Research Center, University of Medicine and Dentistry of New Jersey, 110 Bergen Street, P.O. Box 1709, Newark, NJ 07103 - 2400, USA

## Abstract

Ghrelin, a peptide hormone, newly identified in oral mucosal tissues, has emerged recently as an important mediator of the processes of mucosal defense. Here, we report on the mechanism of ghrelin protection against ethanol cytotoxicity in rat sublingual salivary gland cells. The protective effect of ghrelin was associated with the increase in NO and PGE2, and upregulation in cytosolic phospholipase A_2_ (cPLA_2_) activity and arachidonic acid (AA) release. The loss in countering effect of ghrelin occurred with cNOS inhibitor, L-NAME, as well as indomethacin and COX-1 inhibitor, SC-560, while COX-2 inhibitor, NS-398, and iNOS inhibitor, 1400W, had no effect. The effect of L-NAME was reflected in the inhibition of ghrelin-induced cell capacity for NO production, cPLA_2_ activation and PGE2 generation, whereas indomethacin caused only the inhibition in PGE2. Moreover, the ghrelin-induced up-regulation in AA release was reflected in the cPLA_2_ phosphorylation and S-nitrosylation. Inhibition in ghrelin-induced S-nitrosylation was attained with L-NAME, whereas the ERK inhibitor, PD98059, caused the blockage in cPLA_2_ protein phosphorylation as well as S-nitrosylation. Thus, ghrelin protection of salivary gland cells against ethanol involves cNOS-derived NO induction of cPLA_2_ activation through S-nitrosylation for the increase in AA release at the site of COX-1 action for PGE2 synthesis.

## 1. Introduction

Alcohol abuse is a well-recognized cause of damage to the liver, brain and gastrointestinal tract, and its excessive consumption is associated with an increased risk of cancer of the liver, pharynx, larynx, esophagus, and the oral cavity [1–3]. Moreover, alcoholics and animals exposed to ethanol exhibit diminished secretion of saliva, and often develop inflammation of oral mucosa [3, 4]. The salivary gland acinar cell responses to ethanol cytotoxicity are manifested by the elevation in proinflammatory cytokine production, enhancement in apoptosis, disturbances in nitric oxide (NO) signaling pathways, and the impairment in prostaglandin generation [5–7]. The disturbances in salivary gland acinar cells by ethanol also affect the production of salivary mucins, the glycoproteins that play major role in the preservation of oral mucosal integrity [8, 9]. 

Although the maintenance of mucosal integrity along the alimentary tract relays on multiple molecular processes, the two of the most prominent are the production of NO by the nitric oxide synthase (NOS) system and the formation of prostaglandins generated from AA by the action of cyclooxygenase (COX) enzymes [10–14]. Furthermore, the literature data support the existence of a functional relationship between the products of NOS and COX systems, and there are strong indications that the enzyme compartmentalization and substrate availability determines the segregated utilization of the respective products in physiological and pathophysiological processes [15–17]. Indeed, the stimulation of NO production through NOS induction or the exogenous NO donors leads to up-regulation in COX enzymes activation and the increase prostaglandin, while the inhibition of NOS decreases prostaglandin formation [16–20]. Moreover, the NO-induced COX-2 activation has been linked to the enzyme protein S-nitrosylation [17]. 

Studies indicate that the critical event responsible for rapid changes in prostaglandin production is the release of AA from membrane phospholipids by the action of cytosolic phospholipase A_2_ (cPLA_2_) enzyme [21–23]. The cleavage of AA from *sn-2* position of membrane glycerophospholipids by the action of highly selective Group IV cPLA_2_ is the initial and rate limiting event in prostaglandin production, as well as a key step in the generation of other potent lipid messengers, such as leukotrienes and PAF [22, 24]. The activity of cPLA_2_ is tightly regulated by posttranslational mechanism involving MAPK/ERK-dependent enzyme protein phosphorylation that facilitates the enzyme translocation from cytosol to membrane to gain access to phospholipid substrates [14, 25–27]. Moreover, it has been reported that the NO-induced enzyme protein S-nitrosylation results in cPLA_2_ activation and up-regulation in arachidonic acid release for prostaglandin synthesis [28].

Advances in understanding the nature of factors involved in the maintenance of mucosal integrity along the alimentary tract have brought to the forefront the role of ghrelin in the process of mucosal defense and repair [29–32]. This 28-amino acid peptide hormone, produced mainly in the stomach [29], but also identified recently in oral mucosa, saliva, and the acinar cells of salivary glands [33], has been recognized as an important regulator of the NOS and COX systems, and implicated in the control of local inflammations, healing of experimentally induced gastric ulcers, and the protection of gastric mucosa against acute damage induced by ethanol [30–32, 34, 35].

In this study, we investigated the mechanism of ghrelin protection against ethanol cytotoxicity in the acinar cells of rat sublingual salivary gland. The results of our findings show that ghrelin modulation of ethanol cytotoxicity involves cNOS-derived NO induction of cPLA_2_ activation through S-nitrosylation for the increase in AA release for prostaglandin synthesis.

## 2. Material and Methods

### 2.1. Sublingual Gland Cell Preparation

The acinar cells of sublingual salivary gland were collected from freshly dissected rat salivary glands [14]. The minced tissue was suspended in five volumes of ice-cold Dulbecco's modified (Gibco) Eagle's minimal essential medium (DMEM), supplemented with fungizone (50 *μ*g/ml), penicillin (50 U/ml), streptomycin (50 *μ*g/ml), and 10% fetal calf serum, and gently dispersed by trituration with a syringe, and settled by centrifugation. Following three consecutive rinses with DMEM, the cells were resuspended in the medium to a concentration of 2 × 10^7^ cell/ml. The viability of cell preparations before and during the experimentation, assessed by Trypan blue dye exclusion assay, was greater than 98%.

### 2.2. Ethanol-Induced Cytotoxicity

Aliquots of cell suspension (1 ml) were transferred to DMEM in culture dishes and incubated for 2 hours at 37°C under 95% O_2_/5% CO_2_ atmosphere in the absence and the presence of 3% of ethanol. In the experiments evaluating the effect of ghrelin (rat, Sigma), indomethacin, COX-1 inhibitor, SC-560, COx-2 inhibitor, NS-398 (Sigma), cNOS inhibitor, L-NAME and its inactive isomer, D-NAME, iNOS inhibitor, 1400W and ERK1/2 inhibitor PD98059 (Calbiochem), and ascorbate (Sigma), the cells were first treated for 30 min with the indicated dose of the agent or vehicle followed by 2 h incubation with ethanol [14]. At the conclusion of incubation, the aliquots of cell suspension from the control and various experimental conditions were centrifuged at 300 × g for 5 min and the supernatants used for the measurement of cytotoxicity using TOX-7 lactate dehydrogenase assay kit in accordance with the manufacturer's (Sigma) instructions.

### 2.3. PGE_2_ and NO Quantification

The aliquots of the acinar cell suspension from the control and various experimental conditions were centrifuged at 1500 × g for 5 min and the conditioned medium supernatant collected. PGE_2_ assays were carried out using a PGE_2_ EIA kit (Cayman) and 100 *μ*l aliquots of the spent medium supernatant, according to the manufacturer's instruction. To assess NO production in the acinar cells, we measured the stable NO metabolite, nitrite, accumulation in the culture medium using Griess reaction [36].

### 2.4. AA Release and cPLA_2_ Activity Assay

To assess the release of AA from the acinar cells of salivary gland into the incubation medium, aliquots of the cell suspension (1 ml) were labeled in DMEM with 20 *μ*Ci of [5,6, 8,9, 11,12,14,15-^3^H]arachidonic acid for 4 h [27], and resuspended in fresh DMEM free of albumin. The cells were then treated with the indicated dose of the agent of interest or vehicle and incubated for 2 h in the presence of 3% ethanol, and following centrifugation the supernatant was analyzed for the released [^3^H]arachidonic acid by scintillation spectrometry. The measurement of cPLA_2_ activity in the acinar cells following various experimental conditions was carried out using cPLA_2_ assay kit (Cayman) with thioarachidonoylphosphatidylcholine as substrate [27]. 

### 2.5. cPLA_2_ S-nitrosylation Assay

Detection of cPLA_2_ S-nitrosylation was carried out utilizing a biotin switch procedure for protein S-nitrosylation [37, 38]. The acinar cells were treated with ghrelin (0.7 *μ*g/ml) or L-NAME (400 *μ*M) + ghrelin or PD98059 (30 *μ*M) + ghrelin and incubated for 2 h in the presence of 3% ethanol. Following centrifugation, the recovered cells were lysed in HEN lysis buffer and the unnitrosylated thiol groups were blocked with S-methyl methanethiosulfonate reagent [38]. The proteins were precipitated with acetone, resuspended in HEN buffer containing 1% SDS, and subjected to targeted nitrothiol group reduction with sodium ascorbate (100 mM). The free thiols were then labeled with biotin and the biotinylated proteins were recovered on streptavidin beads. The formed streptavidin bead-protein complex was washed with neutralization buffer, and the bound proteins were dissociated from streptavidin beads with 50 *μ*l of elution buffer (20 mM HEPES, 100 mM NaCl, 1 mM EDTA, pH 7.7) containing 1% 2-mercaptoethanol [37]. The obtained proteins were then analyzed by Western blotting. 

### 2.6. Western Blot Analysis

The acinar cells from the control and experimental treatments were collected by centrifugation, washed with phosphate-buffered saline and resuspended in ice-cold lysis buffer [14]. Following brief sonication, the cell lysates were centrifuged at 12,000 g for 10 min, and the supernatants were subjected to protein determination using BCA protein assay kit (Pierce). The samples, including those from biotin switch procedure, were then resuspended in loading buffer, boiled for 5 min, and subjected to SDS-PAGE using 50 *μ*g protein/lane [27]. The separated proteins were transferred onto nitrocellulose membranes, blocked with 5% skim milk, and incubated with the antibody against the phosphorylated cPLA_2_ protein at 4°C for 16 h. After 1 h incubation with the horseradish peroxidase-conjugated secondary antibody, the phosphorylated proteins were revealed using an enhanced chemiluminescence detection kit (Pierce). Membranes were stripped by incubation in 1 M Tris-HCl (pH 6.8), 10% SDS, and 10 mM dithiotreitol for 30 min at 55°C, and reprobed with antibody against total cPLA_2_. Immunoblotting was performed using specific antibodies directed against cPLA_2_ and phospho-cPLA_2_ (Ser^505^) (Cell Signaling).

### 2.7. Data Analysis

All experiments were carried out using duplicate sampling and the results are expressed as means ± SD. Analysis of variance (ANOVA) followed by nonparametric Kruskal-Wallis test was used to determine significance and the significance level was set at *P* < .05.

## 3. Results

**Figure 1:**
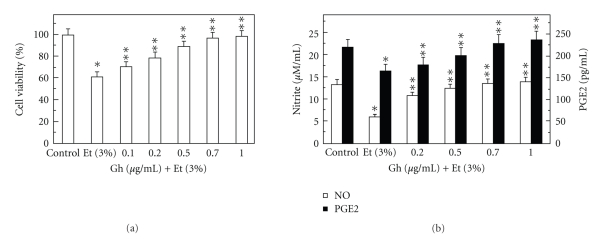
Effect of ghrelin on ethanol-induced cytotoxicity (a) and changes in the production of nitrite and PGE2 (b) in rat sublingual salivary gland acinar cells. The cells, preincubated with the indicated concentrations of ghrelin (Gh), were incubated for 2 h in the presence of 3% ethanol (Et). Values represent the means ± SD of five experiments. *P < .05 compared with that of control. **P < .05 compared with that of Et alone.

To examine the role of salivary ghrelin in oral mucosal protection against ethanol cytotoxicity, we employed primary culture of rat sublingual salivary gland acinar cells exposed to incubation with ethanol in conjunction with lactate dehydrogenase assay [14]. Using ethanol at the dose range (3%) that impairs the cell capacity for mucin synthesis and prostaglandin generation [5, 9], we determined that preincubation of the acinar cells with ghrelin led to a concentration-dependent prevention of ethanol cytotoxicity, and resulted nearly complete protection at 0.7 *μ*g/ml of ghrelin (Figure 1(a)). Moreover, we found that cytotoxicity induced in sublingual salivary gland acinar cells by 3% ethanol was reflected in a 54.5% drop in NO production and a 24.7% reduction in PGE2 generation (Figure 1(b)), and that ghrelin at the concentration of 0.7 *μ*g/ml for the protection against ethanol cytotoxicity evoked a 38.3% increase in the mucosal cell PGE2 generation and a 2.3-fold increase in NO production (Figure 1(b)).

**Figure 2:**
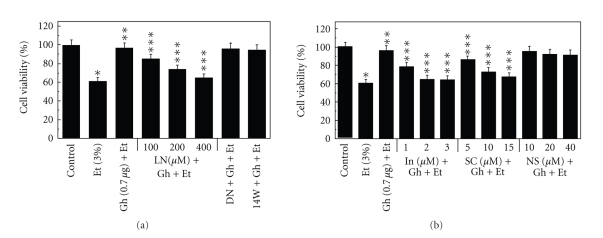
Effect of nitric oxide synthase (a) and cyclooxygenase (b) inhibitors on the ghrelin (Gh)-induced protection of sublingual salivary gland acinar cells against ethanol (Et) cytotoxicity. The cells, preincubated with the indicated concentrations of L-NAME (LN), 200 *μ*M D-NAME (DN) and 30 *μ*M 1400W (14W) or indomethacin (In), SC-560 (SC) and NS-398 (NS), were treated with Gh at 0.7 *μ*g/ml and incubated for 2 h in the presence of 3% Et. The cell-free aliquots of the medium were assayed for lactate dehydrogenase release. Values represent the means ± SD of five experiments. *P < .05 compared with that of control. **P < .05 compared with that of Et alone. ***P < .05 compared with that of Gh + Et.

Our results furthermore revealed that a concentration-dependent loss in the protective effect of ghrelin on the ethanol-induced salivary gland acinar cell toxicity was attained with cNOS inhibitor, L-NAME (Figure 2(a)) as well as cyclooxygenase (COX-1 and COX-2) inhibitor, indomethacin, and a specific COX-1 inhibitor, SC-560 (Figure 2(b)), while selective iNOS inhibitor, 1400W and a specific COX-2 inhibitor, NS-398 had no effect (Figure 2).

**Figure 3:**
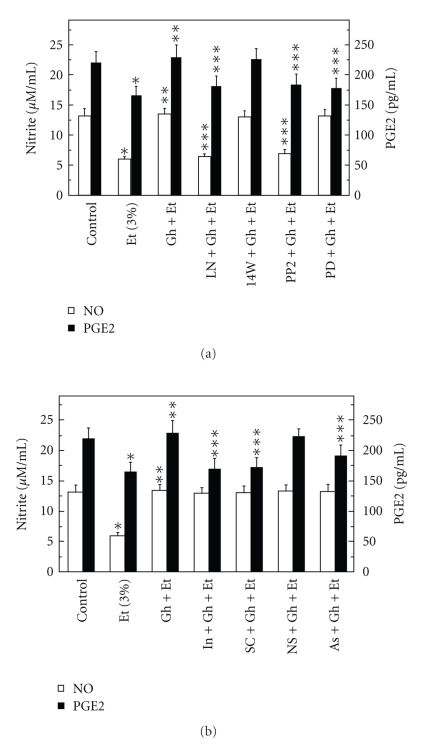
Effect of nitric oxide synthase (a) and cyclooxygenase (b) inhibitors on the ghrelin (Gh)-induced changes in the production of PGE2 and nitrite by sublingual salivary gland acinar cells in the presence of ethanol (Et). The cells, preincubated with 400 *μ*M L-NAME (LN), 30 *μ*M 1400W (14W), 20 *μ*M PP2 and 30 *μ*M PD98059 (PD) or 2 *μ*M indomethacin (In), 15 *μ*M SC-560 (SC), 20 *μ*M NS-398 (NS) and 300 *μ*M ascorbate (As), were treated with 0.7 *μ*g/ml Gh and incubated for 2 h in the presence of 3% Et. Values represent the means ± SD of five experiments. *P < .05 compared with that of control. **P < .05 compared with that of Et alone. ***P < .05 compared with that of Gh + Et.

Moreover, while the effect of L-NAME was reflected in the inhibition of ghrelin-induced acinar cell capacity for NO production as well as PGE2 generation (Figure 3(a)), the pretreatment with indomethacin and COX-1 inhibitor, SC-560, led only to the inhibition in ghrelin-induced PGE2 generation (Figure 3(b)). The stimulatory effect of ghrelin on the acinar cell capacity for NO and PGE2 production, however, was not affected by the inclusion of iNOS inhibitor 1400W and COX-2 inhibitor, NS-398. These results indicate that ghrelin-induced up-regulation in NO production and PGE2 generation occurs with the involvement of respective cNOS and COX-1 enzymes, and suggest the participation of cNOS in sublingual salivary gland acinar cell processes of PGE2 generation in response to ghrelin. 

Further, we found that the countering effect of ghrelin on the ethanol-induced changes in the acinar cell production of NO and PGE2 was subject to suppression by PP2, a selective inhibitor of tyrosine kinase Src (Figure 3(a)). We also revealed that the effect of ghrelin on the acinar cell capacity for PGE2 generation was inhibited by MAPK/ERK1/2 inhibitor, PD98059, whereas the production of NO remained unaffected (Figure 3(a)). These results, thus, implicate the activation of tyrosine kinase Src as a triggering event whereby ghrelin is capable of affecting the acinar cell capacity for NO as well as PGE2 generation. The findings also point to the role of MAPK/ERK in the processes of PGE2 generation.

**Figure 4:**
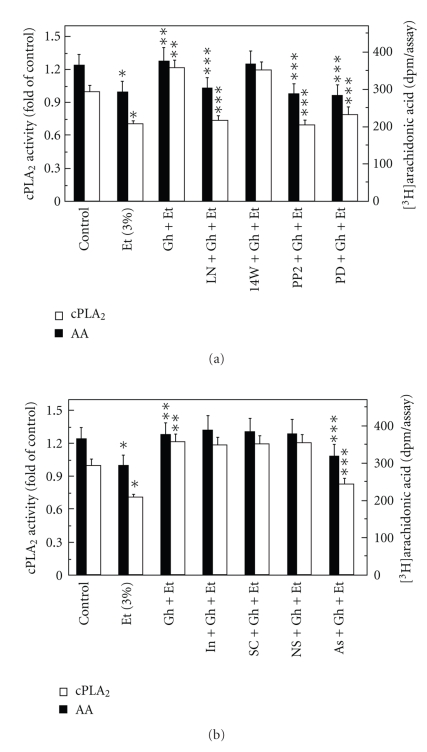
Effect of nitric oxide synthase (a) and cyclooxygenase (b) inhibitors on the ghrelin (Gh)-induced changes in the release of arachidonic acid and cPLA_2_ activity in sublingual salivary gland acinar cells in the presence of ethanol (Et). The cells, preincubated with 400 *μ*M L-NAME (LN), 30 *μ*M 1400W (14W), 20 *μ*M PP2 and 30 *μ*M PD98059 (PD) or 2 *μ*M indomethacin (In), 15 *μ*M SC-560 (SC), 20 *μ*M NS-398 (NS) and 300 *μ*M ascorbate, were treated with Gh at 0.7 *μ*g/ml and incubated for 2 h in the presence of 3% Et. Values represent the means ± SD of five experiments. *P < .05 compared with that of control. **P < .05 compared with that of Et alone. ***P < .05 compared with that of Gh *+* Et.

We next sought additional leads into the involvement of cNOS in ghrelin-induced signaling leading to up-regulation in salivary gland cell PGE2 generation. As the initial and rate limiting step in prostaglandin production is the liberation of arachidonic acid from membrane phospholipids by highly selective cPLA_2_ [22–24], we employed the acinar cells labeled with [^3^H]arachidonic acid to assess the effect of ghrelin on arachidonic release in the presence of nitric oxide synthase inhibition. As shown in Figure 4(a), the ethanol-induced cytotoxicity was reflected in a 20.4% decrease in the acinar cell arachidonic acid release, while preincubation with ghrelin, at its optimal concentration (0.7 *μ*g/ml) for the suppression of the cytotoxic effect of ethanol, resulted in a 28.3% stimulation in arachidonic acid release. This effect of ghrelin on was subject to inhibition by the cNOS inhibitor, L-NAME, while the iNOS inhibitor, 1400W had no effect. Moreover, the ghrelin-induced up-regulation in the acinar cell arachidonic acid release was inhibited by Src kinase inhibitor, PP2 and MAPK/ERK1/2 inhibitor, PD98059 (Figure 4(a)) The stimulatory effect of ghrelin on the arachidonic acid release, however, was not affected by the inclusion of indomethacin or selective COX-1 and COX-2 inhibitors, SC-560 and NS-398 (Figure 4(b)). 

As the activation of cPLA_2_ for rapid release of arachidonic acid involves Src kinase-dependent MAPK/ERK activation of the enzyme through phosphorylation [14, 25, 27], we further measured the acinar cell cPLA_2_ enzymatic activity. We found that preincubation with ghrelin countered the detrimental effect of ethanol on arachidonic acid release and evoked a 71.8% increase in the cPLA_2_ activity (Figure 4). The ghrelin-induced up-regulation in cPLA_2_ activity, furthermore, was subject to suppression by Src inhibitor, PP2 and ERK1/2 inhibitor, PD98059 as well as to the inhibitor of cNOS, L-NAME (Figure 4(a)), whereas pretreatment with indomethacin or selective COX-1 and COX-2 inhibitors did not cause any discernible alteration in the enzyme activity (Figure 4(b)). These results, together with the inhibitory effect of PP2 on the ghrelin-induced increase in NO production (Figure 3(a)), point to the Src kinase as an upstream effector of cNOS in the observed up-regulation in cPLA_2_ activation for the increase in PGE2 generation.

**Figure 5:**
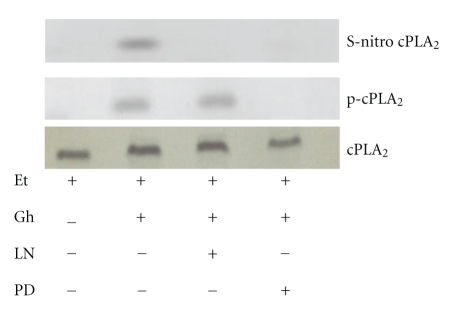
Effect of cNOS inhibitor, L-NAME (LN), and ERK1/2 inhibitor, PD98059 (PD), on ghrelin (Gh)-induced cPLA_2_ S-nitrosylation in sublingual salivary gland acinar cells exposed to ethanol (Et). The cells were treated with Gh (0.7 *μ*g/ml) or L-NAME (400 *μ*M) + Gh or PD98059 (30 *μ*M)+Gh and incubated for 2 h in the presence of Et. A portion of the cell lysate was processed by biotin switch procedure for protein S-nitrosylation and, along with the reminder of the lysates, subjected to SDS-PAGE, transferred to nitrocellulose and probed with phosphorylation specific cPLA_2_ (p-cPLA_2_) antibody, and reprobed with antitotal cPLA_2_ antibody. The immunoblots shown are representative of three experiments.

Recent literature data indicate that up-regulation in NO production exerts the modulatory effect on PGE2 synthesis through protein cysteine S-nitrosylation of cyclooxygenase and cPLA_2_ enzymes [17, 20, 28]. As the S-nitrosylated proteins show susceptibility to ascorbic acid [17, 37, 38], we analyzed the effect of this agent on the ghrelin-induced changes in the acinar cell capacity for PGE2 generation. The results revealed that preincubation of the acinar cells with ascorbate caused not only the decrease in the ghrelin-induced cell capacity for PGE2 production (Figure 3(b)), but also elicited suppression in the ghrelin-induced arachidonic acid release and the activity of cPLA_2 _(Figure 4(b)). To assess further the role of S-nitrosylation in the course of events leading to cPLA_2_ activation by ghrelin, the acinar cells prior to ghrelin incubation, were pretreated with cNOS inhibitor, L-NAME or ERK1/2 inhibitor, PD98059, and the lysates subjected to biotin switch procedure were examined with antibodies directed against phospho-cPLA_2_ and total cPLA_2_. We observed that ghrelin prevention of the ethanol-induced cytotoxicity was reflected in the increase in cPLA_2_ protein phosphorylation as well as S-nitrosylation (Figure 5). Preincubation with L-NAME resulted in the blockage of the ghrelin-induced S-nitrosylation, but had no effect on cPLA_2_ phosphorylation, whereas ERK1/2 inhibitor, PD98059, caused the blockage in cPLA_2_ protein phosphorylation as well as S-nitrosylation. These data demonstrate that the activation of cPLA_2_ in sublingual salivary gland acinar cells by ghrelin involves both the phosphorylation and S-nitrosylation events, and that the enzyme protein phosphorylation is a prerequisite for its S-nitrosylation.

## 4. Discussion

Investigations into the nature of factors involved in the maintenance of mucosal integrity along the alimentary tract, including that of oral cavity, have brought to the forefront the role of ghrelin in processes of mucosal defense and repair [29–33]. This 28-amino acid hormone, produced predominantly in the stomach, but also identified in oral mucosa and the acinar cells of salivary glands [29, 33], has emerged as an important regulator of the cross-talk between NOS and COX enzyme systems, the products of which (NO and PGE2) play direct cytoprotective role in maintaining the soft oral tissue integrity. Moreover, there are reports indicating that NO and PGE2 are involved in ghrelin-induced protection of gastric mucosa against injury by ethanol [31, 32, 34]. 

As the diminished secretion of saliva and oral mucosal inflammatory changes are well-recognized consequences of alcohol abuse on the health of oral cavity [2–4], in the study presented herein we examined the mechanism of ghrelin protection of salivary gland secretory cells against ethanol cytotoxicity. Using the acinar cells of rat sublingual salivary gland exposed to ethanol at the concentration range that impairs mucosal cell capacity for mucin synthesis and prostaglandin generation [5, 9], we demonstrated that the protective effect of ghrelin was associated with the increase in NO and PGE2 production, and marked up-regulation in cPLA_2_ activity and AA release. Moreover, a significant loss in the countering effect of ghrelin on the ethanol-induced toxicity was attained with cNOS inhibitor, L-NAME as well as indomethacin and a specific COX-1 inhibitor, SC-560, while specific COX-2 inhibitor, NS-398, and a selective iNOS inhibitor, 1400W had no effect. These results, are thus in keeping with the literature data demonstrating that the detrimental effects of ethanol on gastric mucosal integrity are associated with the impairment in NO synthesis and PGE2 generation controlled by cNOS and COX-1 enzyme systems [31, 33]. Moreover, our findings on the inhibition of ghrelin-induced acinar cell capacity for NO production as well as PGE2 generation by L-NAME, and only that of PGE2 by indomethacin and COX-1 inhibitor, SC-560, suggest that cNOS-derived NO participates in the regulation of PGE2 production in response to ghrelin.

Since the initial and rate limiting event in prostaglandin production is the release of AA from membrane phospholipids by highly selective cPLA_2_ [22–24], we further assessed the influence of ghrelin on the processes of cPLA_2_ activation. Employing the acinar cell labeled with AA, we demonstrated that ethanol cytotoxicity was manifested in a diminished AA release, while preincubation with ghrelin led to cPLA_2_ activation as evidenced by the increase in AA release. This stimulatory effect of ghrelin was subject to suppression by cNOS inhibitor, L-NAME but not the iNOS inhibitor, 1400W or the inhibitors of COX-1 and COX-2 enzymes. Moreover, we found that up-regulation in AA release and cPLA_2_ activity evoked by ghrelin was also suppressed by the inhibitor of ERK1/2, PD98059. Hence, the ghrelin-induced acinar cell cPLA_2_ activation for the increase in PGE2 production to counter ethanol cytotoxicity requires cNOS and MAPK/ERK participation. This interpretation of our results is supported by the literature data indicating that activation of cPLA_2_ for rapid increase in AA release and eicosanoid generation occurs through posttranslational MAPK/ERK-dependent enzyme protein phosphorylation on the critical Ser^505^ residue that plays a crucial role in Ca^2+^-dependent translocation of cPLA_2_ from cytosol to membrane to gain access to phospholipid substrates [14, 25–27]. 

Interestingly, recent evidence indicates that in addition to posttranslational activation by phosphorylation, the increase in prostaglandin formation may also result from NO-induced enzyme protein S-nitrosylation [17, 20, 28]. Indeed, the posttranslational modification of the protein through S-nitrosylation at the critical cysteine^526^ residue has been linked to the NO-induced enhancement in catalytic activity of COX-2 [17], and cPLA_2_ activation through S-nitrosylation at cysteine^152^ was reported to be responsible for up-regulation in AA release in human epithelial cells [28]. Therefore, to assess the role of cNOS-derived NO in the ghrelin-induced cPLA_2_ activation for the protection of salivary gland acinar cells against cytotoxic effect of ethanol, we examined the cPLA_2_ activity, and its protein S-nitrosylation and phosphorylation. We found that, In keeping with well known susceptibility of S-nitrosylated proteins to reduction by ascorbic acid [17, 37, 38], the ghrelin-induced regulation in cPLA_2_ activity and AA release was liable to suppression by ascorbate. Preincubation with cNOS inhibitor, L-NAME led to the blockage in ghrelin-induced cPLA_2_ protein S-nitrosylation but had no effect on its phosphorylation, whereas ERK1/2 inhibitor, PD98059 caused the blockage in both the cPLA_2_ protein phosphorylation and S-nitrosylation. Thus, the activation of cPLA_2_ in the acinar cells by ghrelin for the increase in AA release and PGE2 synthesis involves the enzyme processing through the events of phosphorylation and S-nitrosylation. It is also apparent that the cPLA_2_ phosphorylation is a prerequisite for its S-nitrosylation.

While our results on the mechanism of ghrelin protection of salivary gland acinar cells against ethanol cytotoxicity are obtained employing the in vitro system and pharmacological concentrations of the peptide, it should be noted that protective effects of the peripherally administered ghrelin against acute gastric mucosal injury induced in rat stomach by ethanol also required considerably higher concentrations of the peptide than that of central ghrelin [30, 32, 34]. This is consistent with the advocated role of hypothalamic neuromodulatory pathways in ghrelin action [39].

In summary, our findings demonstrate that ghrelin protection of salivary gland acinar cells against ethanol cytotoxicity involves cNOS-derived NO induction of cPLA_2_ activation through S-nitrosylation for the increase in PGE2 generation.
